# Transcriptome Analysis Reveals That *FvPAP1* Genes Are Related to the Prolongation of Red-Leaf Period in *Ficus virens*

**DOI:** 10.3390/cimb46060343

**Published:** 2024-06-08

**Authors:** Qingchao Ma, Shuhua Zhong, Tianci Ma, Yajie Yue, Shihui Zou, Shunzhao Sui, Lijiao Ai, Yulong Guo

**Affiliations:** 1 Chongqing Key Laboratory of Germplasm Innovation and Utilization of Native Plants, Chongqing Landscape and Gardening Research Institute, Chongqing 400715, China; mqcmadman@email.swu.edu.cn (Q.M.);; 2 Chongqing Engineering Research Center for Floriculture, College of Horticulture and Landscape, Southwest University, Chongqing 401329, China; zsh15220@email.swu.edu.cn (S.Z.); yyj990928@email.swu.edu.cn (Y.Y.); sszcq@swu.edu.cn (S.S.)

**Keywords:** *FvPAP1*, anthocyanin, *Ficus virens*, red-leaf period, transcriptome analysis

## Abstract

*Ficus virens* is a deciduous tree that is highly valuable both economically and medicinally. Like other plants with ‘red young leaves’, the red-leaf period of most *F. virens* trees lasts only a few days, and the red leaves have little ornamental value. However, in recent years, some lines of *F. virens* with bright red young leaves and a prolonged red-leaf period have been utilized for urban greening. To explore the mechanism of the different lengths of the duration of *F. virens* leaves, we analyzed the physiology and changes in gene expression during the development of two varieties of leaves. The detection of anthocyanin in different developmental stages of the *F. virens* leaves showed that the changes in color of the red leaves of *F. virens* were primarily caused by the change in anthocyanin content. A transcriptome analysis showed that the expression of genes related to the biosynthesis of anthocyanin changed significantly during the development of leaves. A *MYB* gene *FvPAP1*, which was consistent with the change in anthocyanin content, was identified. A real-time quantitative reverse transcription PCR analysis and heterologous expression transgenic studies showed that *FvPAP1* promoted the biosynthesis of anthocyanins. The difference in the expression of *FvPAP1* in time and intensity in the young leaves may be the reason for the difference in the duration of the red-leaf period in different lines of *F. virens*. A sequence analysis showed that the cDNA sequence of *FvPAP1* was polymorphic, and possible reasons were discussed. These results can provide insight for similar studies on the mechanism of the formation of red coloring in other woody plant leaves and provide molecular targets to breed new materials with more prolonged red-leaf periods in *F. virens*.

## 1. Introduction

*Ficus virens* is a deciduous tree that is a member of the Moraceae [1]. It is native to south and southwestern China, particularly in Chongqing, Sichuan, Hubei, and other places, and it is the city tree of Chongqing. *F. virens* is highly valuable both medicinally and ecologically [2,3,4,5,6,7,8]. It is commonly used in roadside afforestation, vertical greening, riverbank protection, and bonsai gardening [9,10,11,12]. Many plants have young red leaves [13], which is a phenomenon known as the ‘red young leaves’. Since the young leaves of most plants rapidly turn green as they develop, and the red color simultaneously disappears, the short red-leaf period has no obvious ornamental value [14]. However, some varieties of these plants that remain red for longer periods have also been isolated and propagated, including *Photinia × fraseri* [15], *Prunus cerasifera* [16], *Populus deltoids* [17], ‘Red Dragon’ *Corylus avellana* [18], and *Cornus florida* [19].

The concentrated defoliation of *F. virens* primarily occurs in the spring from March to May in Chongqing. When a *F. virens* tree defoliates, the leaves of the whole tree will quickly fall within a few days. Simultaneously, the new leaves wrapped in the giant stipules will quickly expand, and the new and old leaves will be replaced within a few days [5]. The whole canopy quickly changes from the yellow of the aging leaves to the color of the new leaves, and the entire process is considered to be spectacular. Like most plants with red young leaves, the red-leaf period of *F. virens* only lasts for a short time, which renders the plant relatively invaluable ornamentally. However, with the wide application of *F. virens* in landscaping in recent years, nursery workers have intentionally or unintentionally propagated some lines with obviously prolonged red-leaf periods and enhanced aesthetic value (Figure S1).

The primary material basis for the formation of red leaves is the anthocyanin members of the flavonoids [13]. The genes that encode the anthocyanin biosynthetic enzymes can be divided into early biosynthetic genes (EBGs) and late biosynthetic genes (LBGs) [20]. The EBGs include chalcone synthase (*CHS*), chalcone isomerase (*CHI*), and flavanone 3-hydroxylase (*F3H*), which are located upstream of the anthocyanin biosynthetic pathway. The LBGs include flavonoid 3′-hydroxylase (*F3*′*H*), flavonoid 3′5′-hydroxylase (*F3*′*5*′*H*), dihydroflavonol 4-reductase (*DFR*), and anthocyanin synthase (*ANS*), which are located downstream of the anthocyanin biosynthetic pathway. The EBGs and LBGs are structural genes in the anthocyanin biosynthetic pathway.

The regulatory mechanism of the biosynthesis of anthocyanins in plants is relatively conservative and primarily acts through the MBW complex formed by the combination of the transcriptional regulators MYB, bHLH, and WD40 to bind to the promoter region of each anthocyanin biosynthetic gene to regulate their transcription. In most cases, bHLH and WD40, which constitute the MBW complex, are constitutively expressed. Therefore, the spatiotemporal specificity of the formation of the MBW complex is determined by the specificity of the expression of MYB transcription factors (TFs), which, in turn, determines the spatiotemporal characteristics of anthocyanin biosynthesis [21]. An MYB TF protein is characterized by a highly conserved R domain that binds to DNA. The number of R domains can be divided into four categories, namely, R2R3-MYB, 1R-MYB, 3R-MYB, and 4R-MYB. The MYB activators that regulate anthocyanin biosynthesis are primarily from R2R3-MYB [21,22]. The R2R3-MYB activators, such as *Arabidopsis thaliana* AtMYB75/PAP1, AtMYB90/PAP2, AtMYB113, AtMYB114, PhAN2, and *Petunia hybrida* PhAN4, play a positive regulatory role in the biosynthesis of anthocyanin [23,24,25], while the MYB repressors, including R2R3-MYB (AtMYB3, AtMYB4, AtMYB7, AtMYB32, and PhMYB27) and R3-MYB (AtMYBL2 and PhMYBx) [26,27,28], inhibit anthocyanin biosynthesis by competing with the R2R3-MYB activators.

The prolonging of the red-leaf period would be achieved by enhancing the biosynthesis and/or stability of the anthocyanins in which the enhancement of biosynthesis is the basis. Prolonging the red-leaf period of the young leaves in *F. virens* can enhance its ornamental value. However, to our knowledge, research on the mechanism of red leaf formation in *F. virens* has not yet been reported. Based on the fact that the MYB TFs control the expression of anthocyanin biosynthesis in plants in most cases, we hypothesized that prolonging the red young leaves of *F. virens* may be related to the changes in expression of one or several *MYB* genes. To verify this hypothesis, we conducted a transcriptome sequencing analysis on the wild-type (W-type) and red-type (R-type) leaves of *F. virens* with different lengths of red-leaf periods to explore the changes in gene expression during the development of leaves. The structural and regulatory genes of the anthocyanin biosynthetic pathway were identified by their phylogenetic relationship, and a real-time quantitative reverse transcription PCR (qRT-PCR) determined their trends of expression. The R2R3-MYB gene *FvPAP1*, which may promote anthocyanin biosynthesis, was eventually cloned and functionally verified. The results showed that during the development of *F. virens* leaves, the change in color of the red leaves was caused by a change in the anthocyanin content. The structural genes and regulatory genes related to anthocyanin biosynthesis are involved in regulating the extension of the red-leaf period of *F. virens*. *FvPAP1* can activate the biosynthesis of anthocyanin, which may be related to extending the red-leaf period. These results can provide molecular targets for the selection of *F. virens* plants with a further extension of the red-leaf period.

## 2. Materials and Methods

### 2.1. Plant Materials

To observe and conveniently collect materials, this study was conducted with 2-year-old cuttings approximately 2 m high. They were planted in the Beibei District of Chongqing, China (106°18′02′′~106°40′57′′ E. 29°37′~30°05′08′′ N; average altitude: 358 m) under natural conditions. The contents of anthocyanin and chlorophyll were determined, and the transcriptome sequencing was conducted in April 2022. qRT-PCR was performed in April 2023. After the first young leaf was exposed from the stipules, the young leaves of each branch were numbered from the bottom to top. The third leaves were used as material to determine the contents of anthocyanin and chlorophyll and perform a qRT-PCR analysis.

The *P. hybrida* inbred line ‘Mitchell Diploid’ and *N. tabacum* variety ‘K326’ were used as the transgenic receptors. *P. hybrida* plants for the transient expression assay were grown in a greenhouse (greenhouse model: LB96Ss4, China) with no exogenous heat and light supplementation. Aseptic *N. tabacum* seedlings were cultured under alternating light and dark conditions at 16:8 h (24°:20 °C).

### 2.2. Quantitative Analysis of Anthocyanins

The anthocyanin content was determined by the HCl methanol method [25,29]. A total of 0.25 g fresh sample was ground into powder in liquid nitrogen and incubated in 10 mL 1% HCl–methanol (*v*/*v*) at 4 °C in the dark for 1 h, shaking 2–3 times, 6000/7000 rpm, and 7–10 min centrifugation. A volume of 200 μL of the supernatant was placed in 96-well microtiter plates, and the absorbance of the supernatant was measured at 530 nm and 657 nm on a Varioskan Flash Spectral Scanning Multimode Reader (Thermo Fisher Scientific, Waltham, MA, USA). The relative anthocyanin content was then determined to be (A530 − 0.25 × A657)/W g^−1^ fresh weight.

### 2.3. Quantitative Analysis of Chlorophyll

The chlorophyll content was determined by the acetone ethanol mixing method [30]. A fresh sample of 0.2 g leaves was cut into pieces and placed in a 1:1 ratio of 20 mL acetone–anhydrous ethanol. The mixture was placed at room temperature, and the leaves were soaked for 24 h until they had completely whitened. A volume of 200 μL of the supernatant was placed in a 96-well microtiter plate. The absorbance of the supernatant at 633 nm and 645 nm was measured on the Varioskan Flash Spectral Scanning Multimode Reader. The relative total chlorophyll content was (8.02 × A633 + 20.21 × A645) · 1000 V/W mg fresh weight.

### 2.4. Transcriptomic Analysis

Based on the Illumina NovaSeq 6000 sequencing platform (Illumina, San Diego, CA, USA), the library was constructed using the Illumina TruSeq^TM^ RNA sample prep Kit method. The total RNA was extracted from the leaves of different developmental stages of *F. virens*. The RNA concentration, purity, and integrity were detected by a NanoDrop 2000 (Thermo Fisher Scientific) and agarose gel electrophoresis, and the RIN value was determined by an Agilent 2100 (Agilent Technologies, Santa Clara, CA, USA). The mRNA was isolated from the total RNA using magnetic beads with Oligo (dT). The mRNA was randomly broken by adding fragmentation buffer, and a small fragment of approximately 300 bp was isolated by magnetic bead screening. Under the action of reverse transcriptase, random hexamers were added to reverse the biosynthesis of one-stranded cDNA, followed by two-stranded biosynthesis. End Repair Mix was added to fill it into a flat end, and an A′′ base was then added to the 3′ end to connect the Y-shaped connector. An Illumina platform was used for sequencing.

The transcriptome analysis of 15 samples was completed, and a total of 124.22 Gb Clean Data were obtained. The Clean Data of each sample was >7.04 Gb, and the percentage of Q 30 bases was more than 93.68%. The Clean Reads of each sample were compared with the banyan (*F. benghalensis*) genome sequence, and the alignment rate ranged from 61.8% to 63.51%, which was lower than the generally acceptable 70%. Therefore, we used Trinity (k-mer = 30) to perform the de novo assembly of all the sample clean data and optimize the assembly results.

The level of expression and DEG analysis were based on the default parameters and software of the Meiji Bioplatform [31]. The expression software was RSEM (Version 1.3.1), and the expression index was TPM. The differential expression analysis (DEG) software was DESeq2 (Version 1.38.0), *p*-adjust < 0.05, and the multiple test correction method was BH. The DEG groups were Rs4 vs. Rs5, Rs5 vs. Rs6, Rs4 vs. Rs6, Ws4 vs. Ws5, Rs4 vs. Ws4, and Rs5 vs. Ws5.

The cluster analysis was analyzed in the form of the mean value of the group; the log base value was 10; the gene clustering algorithm was expanded by hierarchical clustering; the gene clustering method was average; and the gene distance algorithm was Euclidean. A cluster analysis divided all the genes into five sub-clusters, which corresponded to heatmap1, heatmap2, heatmap3, heatmap4, and heatmap5.

A WGCNA visual analysis utilized the default module of the Meiji platform. In the module identification part, the networkType was signed; the soft power was 9; minModuleSize was 30; minKMEtoStay was 0.3; and mergeCutHeight was 0.25. In the module analysis, the correlation coefficient was calculated using Spearman; the anthocyanin content in the corresponding period was selected as the phenotypic data; and the phenotypic data type was continuous. The first 30 connectivity nodes in the screening module were analyzed in the visual analysis, and the connections between the nodes with weight values > 0.02 were screened for analysis.

### 2.5. Identification of the Structural Genes of the Anthocyanin Pathway

Since thousands of genes were differentially expressed for the adjacent developmental stages, the structural genes related to anthocyanin biosynthesis were primarily conducted on the basis of KEGG pathway annotation. The genes related to anthocyanin biosynthesis were primarily related to MAP00941 and MAP00942 in the KEGG pathway [32].

### 2.6. Phylogenetic Analysis for the MYB Genes

A phylogenetic analysis using the protein sequences was applied to search for the MYB genes. As previously described, the sequences of 13 MYB proteins related to anthocyanin biosynthesis in *A. thaliana* and petunia were retrieved from GenBank, and their accession numbers were as follows: PhAN2 (BAP28593.1), PhAN4 (WGV46820.1), PhMYB27 (AHX24372.1), PhMYBx (AHX24371.1), AtMYB3 (BAA21618.1), AtMYB4 (BAA21619.1), AtMYB7 (Q42379.1), AtMYB32 (EFH43356.1), AtPAP1/AtMYB75 (Q9FE25.1), AtPAP2/AtMYB90 (Q9ZTC3.1), AtMYB113 (Q9FNV9.1), AtMYB114 (Q9FNV8.1), and AtMYBL2 (NP_001321410.1). Owing to large differences in the C-terminal sequences of MYB proteins even among functionally equivalent members, only the R2R3 domains were used in the MYB phylogenetic analysis. The phylogenetic trees were constructed by MEGA 7 [33]. Parameters for the NJ tree were set as the p-distance model and pairwise deletion with the bootstrap value as 1000.

### 2.7. Primer Design

Considering the potential existence of errors in the contiguous sequences obtained by de novo transcriptome assembly, we decided to confirm the target gene sequences by PCR cloning and sequencing before designing quantitative PCR primers to analyze the changes in gene expression. If a target unigene assembled by Trinity (k-mer = 30) was hypothesized to have a complete CDS sequence, the primers were first designed to amplify its CDS. If the amplification did not produce any bonds, we assumed that the assembly by Trinity (k-mer = 30) should be incorrect, and new primers were designed by re-referring to the other Trinity (k-mer = 25) assembly result and/or the sequence of homologous genes in the genome sequencing results of *F. microcarpa*. If the target unigene assembled by Trinity (k-mer = 30) did not have a complete CDS sequence, the unigene in the Trinity (k-mer = 25) assembly results and the homologous gene of *F. microcarpa* genome were analyzed to search a complete CDS. If the complete CDS was identified, primers were designed based on the corresponding sequences. The primers were designed and evaluated using Primer Premier 6 (http://www.premierbiosoft.com/primerdesign/index.html, accessed on 20 November 2022). The primers used in this study are shown in Table 1.

### 2.8. Cloning of the Transcript Sequences Related to Anthocyanin Biosynthesis

The stage 4 leaves of the R-type and W-type *F. virens* were collected, and the total RNA was extracted using a Boer plant rapid extraction kit (B2114, Chongqing Boer Biotech Co., Ltd., Chongqing, China). The quality of total RNA was measured by agarose gel electrophoresis, and the concentration of RNA was measured by a NanoDrop 2000 spectrophotometer (Gene Company Limited, Beijing, China). The cDNA was synthesized to clone the gene using the All-in-One First-Strand Synthesis MasterMix (with dsDNase) (Cat: EG15133S, Jiangsu Yugong Biotech Co., Ltd., Lianyungang, China). The PCR was amplified using Top Taq enzyme (AP151-11, TransGen Biotech Co., Ltd., Beijing, China). The amplification parameters were adjusted based on the primer Tm value. The amplification product was ligated to T-Vector pMDTM19 (Simple) (3271, Takara Bio Co., Ltd., Shiga, Japan) for Sanger sequencing.

### 2.9. qRT-PCR

A total of 2000 ng was used to synthesize 40 μL of cDNA as a template. Real-time PCR was conducted using 2 × TSINGKE^®^ Master qPCR Mix kit (SYBR Green I with UDG) (TSE203, Beijing Tsingke Biotechnology Co., Ltd., Beijing, China). A volume of 10 μL of reaction system with 1 μL of upstream/downstream primers, and 1 μL of cDNA template was used for each PCR reaction. *Actin* (OR682436) and *Ubiquitin* (OR682446) were used as the internal reference genes in *F. virens*, and the *PhSAND* and *RPS13* genes were used as internal reference genes in the *P. hybrida* material [25]. The primers for quantitative PCR were designed in the 3′ non-conserved region of the CDS. The quantitative PCR primers used in the experiment are shown in Table 1. Quantitative PCR was performed on a CFX96 Optical Reaction Module for Real-Time PCR (2023 Bio-Rad, Hercules, CA, USA). Bio-Rad CFX Manager 3.1 software, and the 2^−ΔCT^ method was used to analyze the gene expression [34].

### 2.10. Construction of the FvPAP1 Expression Vector

The T-Vector plasmid that harbored the complete CDS of *FvPAP1* as a template was used with NocIPAP1-F: catttacgaacgatagccATGGATGGCCGTTCCT and PstIPAP1-R: gctcaccatctgcagactacctccATTTCCTTGGTCGAGATCC to add restriction sites *NocI* and *PstI* to the upstream and downstream of the *FvPAP1* coding region sequence, respectively. The pGMF500 plasmid (Figure S2) and the target fragments were digested with *Pst1* and *Noc1* and ligated. The *FvPAP1* sequence was cloned into the pGMF500 plasmid and placed under the control of the 35S promoter and the NOS terminator. The correct plasmid that was verified by sequencing was designated pGmf-*FvPAP1*. The plasmid was introduced into *Agrobacterium* GV3101 (pSoup) by the heat shock method for plant genetic transformation.

### 2.11. Transient Expression

The *P. hybrida* petals and leaves were transiently expressed using the *Agrobacterium* injection method as described by Yuan Meng et al. [35]. The *Agrobacterium* that harbored pGmf-*FvPAP1* was selected and cloned into 700 μL of YEB liquid medium that contained the corresponding antibiotics and cultured at 28 °C for 24 h at 200 rpm. A volume of 200 μL of the cultured *Agrobacterium* solution was transferred to 20 mL of YEB medium that contained the corresponding antibiotics. The LB medium contained 15 μM of acetosyringone (AS). It was cultured at 28 °C, 200 rpm until the logarithmic phase of *Agrobacterium* growth (OD_600_ = 0.5–0.6). The cells were centrifuged at 7000 rpm for 10 min at room temperature to collect the cells, and the cells were suspended in the infected fluid that contained 10 mM MgCl_2_, 10 mM MES, and 150 μM acetosyringone, pH = 5.6 to OD_600_ = 0.8. It was incubated stationary at room temperature for 2~3 h. A 1 mL syringe was used to gently open a small opening on the back of the petals and leaves of *P. hybrida*, and the bacterial liquid was sucked from the syringe tube that removed the syringe and injected from the wound. The injected plants were placed in the dark for 24 h and cultured at 24:20 °C for 16:8 h.

### 2.12. Genetic Transformation of Nicotiana Tabacum

*N. tabacum* was transformed genetically using the leaf disc method [36]. The seeds were disinfected with 75% ethanol for 30 s and then mixed with 5% sodium hypochlorite (NaCIO) and sterile water at a ratio of 3:1 for 10 min. After disinfection, the *N. tabacum* seeds were washed 4–5 times with sterile water and transferred to MS media. When the *N. tabacum* leaves grew to four or five true leaves, the leaves were cut to 0.5 × 0.5 cm and placed in MS + 2.0 mg/L 6-benzylaminopurine (6-BA) + 0.2 mg/L 1-napthaleneacetic acid (NAA) media for pre-culture for 2 d. The *Agrobacterium* GV3101 (pSoup) transformed with pGmf-*FvPAP1* was cultured in YEB liquid media to OD_600_ = 0.8 and then resuspended in the infection solution (MS + 50 mg L^−1^ AS + 50 mg L^−1^ MES) to OD_600_ = 0.4. The pre-cultured *N. tabacum* leaves were shaken at low speed in the dark for 10 min and transferred to MS + 2.0 mg L^−1^ 6-BA + 0.2 mg L^−1^ NAA + 500 mg L^−1^ Cb + 5 mg L^−1^ Basta media for screening culture.

## 3. Results

### 3.1. Morphological Characteristics of Ficus virens during Leaf Development

**Figure 1:**
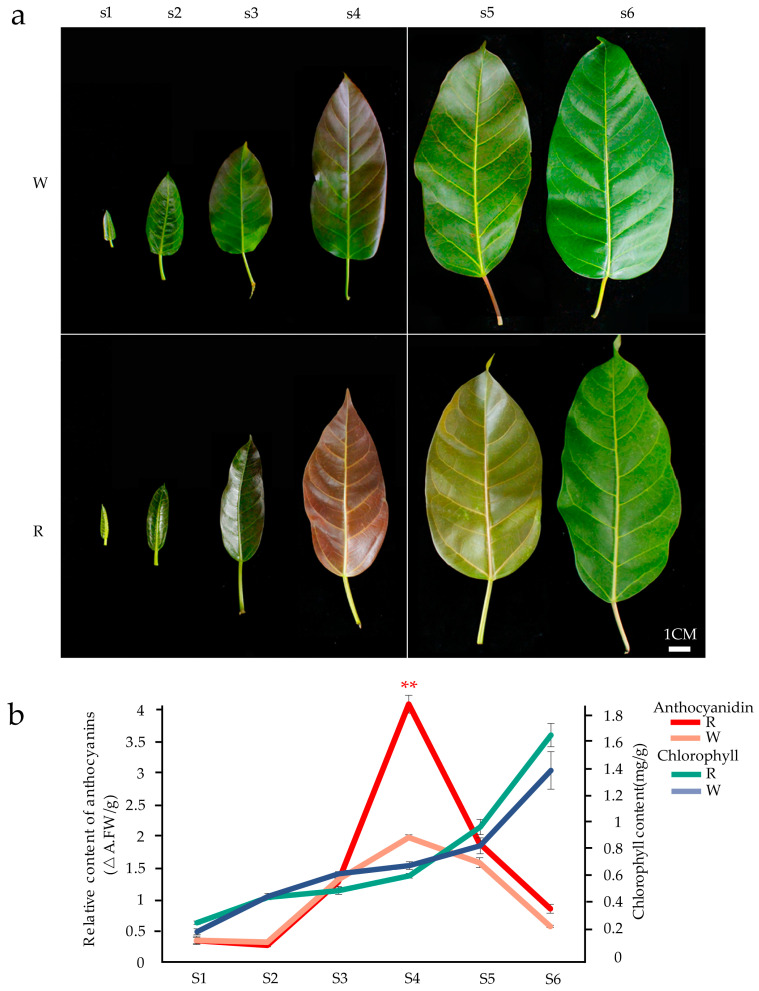
Changes in leaf size and pigment content of two varieties of *Ficus virens* at different developmental stages: (**a**) The morphological changes in the leaves of two types of *F. virens* at different developmental stages. W: wild-type; R: red-type. s1 (stage 1): the stage of bud wrapped by stipules; s2, s3, s4, s5 and s6: the first, third, seventh, thirtieth, and fortieth day after the day of expansion, respectively. Bar = 1 cm. (**b**) The changes in pigment content in the leaves of two kinds of *F. virens* in the sixth stage, including anthocyanin and total chlorophyll. The significance of the difference between adjacent stages was tested by a single-factor analysis of variance and *t*-test. ** *p* < 0.01. In the figure, the anthocyanin content of the W-type and R-type only differed significantly in the s4 period.

During the development of young leaves, the whole bud is usually wrapped by a large stipule until the length of the bud reaches approximately 10 cm, and the true leaf expands and breaks through the outer stipules that usually show different degrees of red (Figure S3). When the true leaves are wrapped and on the day of expansion, they are green. They become obviously red on the third day after expansion, and the red is most obvious on the seventh day after expansion. The redness of the leaves will then gradually fade until the inherent green of the leaves is presented. The new leaves of most *F. virens* plants are light red, and the red-leaf period last for approximately 10 days. Because most *F. virens* trees manifest this red-leaf period, we regard them as the wild-type (W-type) (Figure 1a). In recent years, the new leaves of some *F. virens* trees have been found to be bright red, and the red state lasts for 20–30 days. This type of *F. virens* is called the red-type (R-type) *F. virens* (Figure 1a) in this study. The anthocyanin and chlorophyll of the leaves of these two varieties of *F. virens* were determined at different stages. The results showed that the chlorophyll content increased with the development of the leaves, and the change in the anthocyanin content was consistent with the red change in the leaves that was observed (Figure 1b). When the expanded leaves appeared red (Stage 2), there were significant differences in the anthocyanin content between the two varieties of *F. virens* at adjacent development stages. This demonstrates that the red formation of the leaves of *F. virens* is related to the biosynthesis of anthocyanins.

### 3.2. Comprehensive Transcriptomic Analysis

To explore the key genes in the formation of red leaves and explore the possible mechanism of the elongation of the red-leaf period in *F. virens*, the leaves of s4 and s5 from the W-type plants (Ws4 and Ws5) and s4, s5, and s6 from the R-type plants (Rs4, Rs5, and Rs6) (Figure 1a) were collected for transcriptome sequencing. A transcriptome analysis of 15 samples obtained 124.22 Gb of clean data. Trinity (k-mer = 30) was used to assemble clean data from all the samples, and the assembly results were optimized and evaluated. There were 52,200 unigenes, and they were 1018.03 bp long on average.

**Figure 2:**
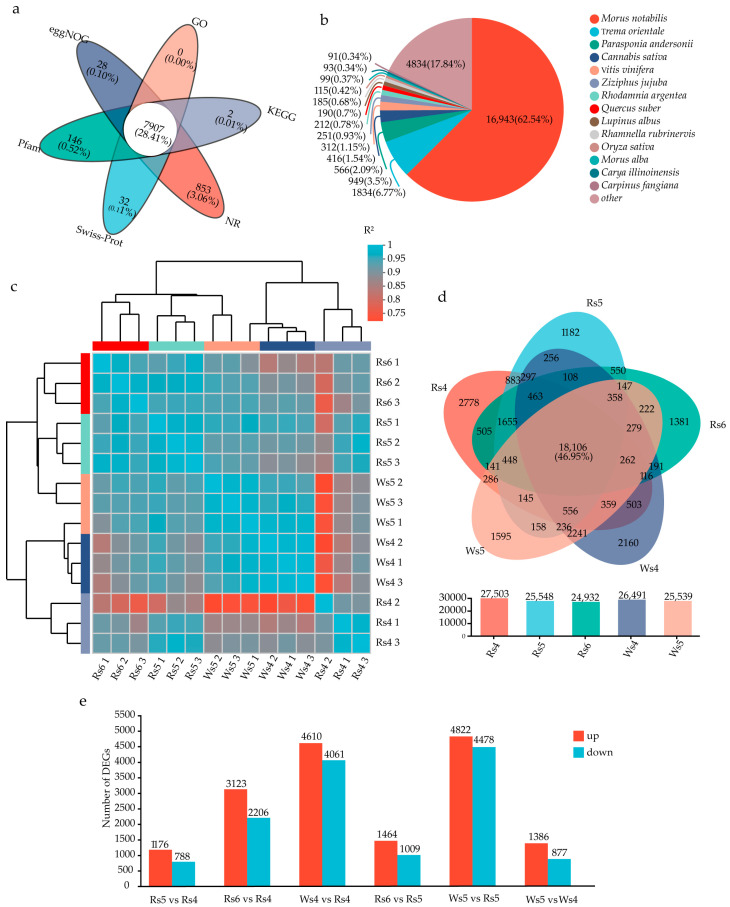
Transcriptome analysis of different leaf development stages of *Ficus virens*: (**a**) Venn diagram of the number of unigenes annotated in the NR, Swiss-Prot, Pfam, COG, GO, and KEGG databases. (**b**) NR annotated species pie chart. (**c**) Sample correlation heat map. The right and lower sides of the map are sample names, and the left and upper sides are sample clustering situations. Color represents the correlation coefficient among samples. (**d**) Venn diagram showing co-expression of genes in different sample. (**e**) Statistical map of the DEGs between samples. The abscissa represents different comparison groups, and the ordinate represents the corresponding number of up- and downregulated genes. COG: Clusters of Orthologous Genes; DEGs: differentially expressed genes; GO: Gene Ontology; KEGG: Kyoto Encyclopedia of Genes and Genomes.

After comparison with the six major databases, there were 23,125, 11,473, 21,758, 27,063, 20,784, and 19,841 unigenes annotated in the NR, Swiss-Prot, Pfam, Clusters of Orthologous Genes (COG), Gene Ontology (GO), and Kyoto Encyclopedia of Genes and Genomes (KEGG) databases, respectively. There were 7907 genes annotated in all six datasets (Figure 2a). A total of 62.54% of the genes annotated in the NR library were from *Morus notabilis* (Figure 2b), which is also a member of the Moraceae family like *F. virens*.

The repeatability correlation among different samples of the leaves of the two lines was reasonable (Figure 2c). For unigenes with expression > 0.1 transcript per million (TPM), 22,397 unigenes were annotated for the W-type, 20,672 for the R-type, and 18,106 unigenes were co-annotated (Figure 2d). A large number of DEGs between any two different stages of leaf samples were detected in this study (Figure 2e).

### 3.3. Identification of the Structural Genes Related to Anthocyanin Biosynthesis and the MYB Genes

Anthocyanins are flavonoids, and the biosynthesis of anthocyanin is primarily regulated owing to the activities of MAP00941 and MAP00942 in the KEGG pathway. Using the results of assembly at k-mer = 30, a total of 85 structural genes related to anthocyanin biosynthesis expressed in the leaves of the wild-type and R-type were obtained by KEGG. Anthocyanin reductase (*ANR*), UDP-glucuronosyltransferase (*UGT*), and *F3H* are single-copy genes, and other structural genes have multiple copies. *ANS* and *F3*′*5*′*H* were not found in the transcriptome database. An analysis of the differentially expressed genes (DEGs) obtained 57 DEGs from the 85 structural genes. We analyzed 141 MYB genes obtained from the transcriptome data and obtained 71 *MYB* DEGs.

**Figure 3:**
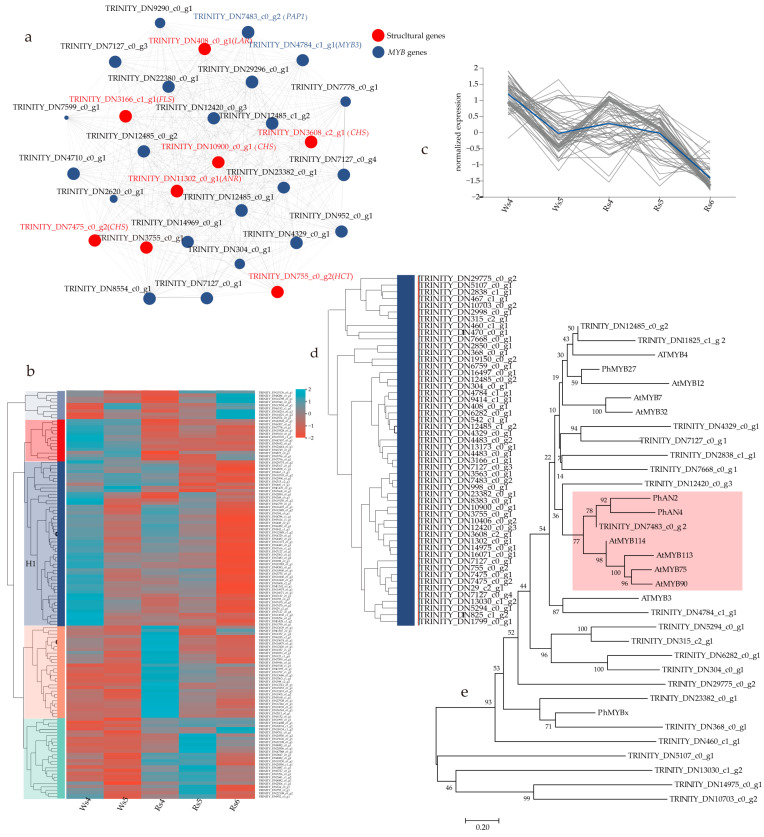
Identification of the structural and regulatory genes related to anthocyanin biosynthesis in *Ficus virens*: (**a**) WGCNA visual network diagram. The correlation among the genes was visualized. Each node in the visualization graph represents a gene. Greater the connectivity of a node indicates more importance. Red is marked as the structural gene related to anthocyanin biosynthesis. (**b**) The clustering heat map of the structural DEGs and regulatory DEGs related to anthocyanin biosynthesis in the transcriptome leaves of *F. virens*. Each column in the figure represents a sample; each row represents a unigene, and the color represents the level of expression of the unigene in the sample. The left side is the tree diagram of unigene clustering. The closeness of two unigene branches indicates closer levels of expression levels. (**c**) Chart of the trend of expression of the sub-cluster heatmap1 genes. Each line (gray) in the figure represents a unigene change trend, and the fitting line (blue) represents the change trend of the average expression of all unigenes in the sub-cluster. (**d**) Sub-cluster heatmap1 detail. (**e**) Phylogenetic analysis of the putative MYB members. WGCNA: weighted gene co-expression network analysis.

Since the activation of LBGs requires the MBW complex [28], we performed a weighted gene co-expression network analysis (WGCNA) visualization and cluster analysis using the 57 DEGs that were shown to encode anthocyanin biosynthetic enzymes and 71 *MYB* DEGs. A WGCNA visualization analysis enabled the identification of the structural genes *CHS*, *ANR*, *F3H*, leucoanthocyanidin reductase (*LAR*), flavonol synthase (*FLS*), and shikimate/quinate hydroxycinnamoyltransferase (*HCT*) that were related to the changes in anthocyanin content. Among them, there were three copies of *CHS*s, and the rest were expressed as one copy (Figure 3a). With the exception of *HCT*, the other genes were the members with the highest level of expression in their respective gene family. A cluster analysis was used to obtain the *MYB* genes that were consistent with the trend of expression of the structural genes related to anthocyanin biosynthesis. The clustering results showed that these genes primarily clustered in the heatmap1 subclass, with a total of 52 genes (Figure 3b). The trend of expression of this subclass was basically consistent with the trend of anthocyanin content in the leaves (Figure 3c). A total of 24 highly expressed anthocyanin structural genes (Table 2) were found in the heatmap1 sub-cluster. These 24 genes contained eight anthocyanin structural genes obtained by WGCNA visualization analysis, which served as the basis for the following *MYB* gene analysis using Heatmap1.

The 28 *MYB* genes that were identified from the Heatmap1 subclass (Figure 3d) were used to construct phylogenetic relationships with the 13 *MYB* genes from *A. thaliana* and *Petunia hybrida*. The results showed that TRINITY_DN7483_c0_g2 clustered with PhAN2 and AtMYB113 activated anthocyanin biosynthesis (Figure 3e). This finding indicated that it may play a role in promoting anthocyanin biosynthesis during the development of *F. virens* leaves. Simultaneously, this gene is also one of the ones related to the change in anthocyanin content identified by the WGCNA visualization analysis (Figure 3a). Therefore, we hypothesized that it may play a role in regulating the red duration of the W-type and R-type *F. virens* leaves. It was further cloned and analyzed together with the eight structural genes (Table 2) obtained from the WGCNA visualization analysis.

### 3.4. Cloning of the Transcript Sequences Related to Anthocyanin Biosynthesis

Considering that the sequences assembled by transcriptome sequencing using next-generation sequencing technology (NGS) may be incorrect and different from the actual sequences, we decided to first clone the complete coding frame of each target gene to more reliably examine the changes in expression and analyze the gene functions. The members with the highest level of expression in each gene related to the anthocyanin biosynthesis family identified by WGCNA and a cluster analysis were selected for gene cloning. The sequences cloned in this study were all transcripts. First, primers were designed based on the sequence assembled by Trinity (k-mer = 30), and their complete coding frame sequences were amplified. The primers used for amplification are shown in Table 1.

The amplification results showed that the amplified fragments of *FvDFR1* (OR682442) and *FvF3*′*H* (OR682443) were consistent with the length of their respective unigene. The assembled *FvBZ1* unigene was 1104 bp long, while the actual amplified fragment was 1410 bp (OR682439). In comparison with other *CHI* genes from plants, the assembled *CHI* unigene only represent a 5′ partial CDS. To clone the complete CDS, we searched the recently published genomic sequence of the closely related plant *Ficus microcarpa* for homologous genes. The 3′ primer was designed based on the homologous reference sequence (GWHPABKV009265.1), and the complete CDS (OR682440) was obtained. The *UGT* gene was not identified by the clustering analysis and WGCNA described above. We chose to clone *UGT* because it was closely related to the stability of anthocyanin. The assembled *UGT* unigene only represents a 5′ partial CDS. The 3′ primers were designed based on the homologous sequence of *F. microcarpa* (GWHPABKV019926.1), and the complete CDS (OR682445) of *UGT* was cloned (Figure S4).

Since the reference sequence obtained by Trinity using the default parameter (k-mer = 30) was not annotated to *ANS* and the other genes, we adjusted the parameter of Trinity (k-mer from 30 to 25) to re-assemble, and K-mer25_TRINITY_DN2320_c1_g1 was annotated as an *ANS* gene. The product that was obtained by PCR amplification using primers based on this sequence was consistent with the predictions (OR682438, Figure S4).

The PCR product obtained using primers designed based on the assembled *CHS* unigene sequence was substantially different from the unigene sequence (homology < 27%). However, the 195 bp PCR products that were amplified with quantitative primers FvCHS1-QF and FvCHS1-QR were consistent with the TRINITY_DN3608_c2_g1 unigene. The primers designed based on TRINITY_DN10900_c0_g1 did not amplify any product. Thus, it is likely that the assembled sequence was incorrect. Since the complete CDS of the candidate CHS genes were not cloned according to the sequence assembled by Trinity (k-mer = 30), we re-designed the PCR primers based on the highest expressed *CHS* sequence K-mer25_TRINITY_DN579_c1_g1 assembled by the parameter-adjusted Trinity (k-mer = 25) method. The cloning and sequencing of the amplified product showed that it was consistent with the unigene (Figure S4), and we designed it as *FvCHS2* (OR682441).

Only one unigene represented *F3H* in the Trinity (k-mer = 30)-assembled results, but no product was amplified using the primers designed based on the unigene sequence. However, a 179 bp fragment was produced with the real-time PCR primers *FvF3H*-QF and *FvF3H*-QR designed according to the unigene sequence, and its sequence was consistent with the origin unigene (Figure S4).

An analysis of the putative protein sequence of the TRINITY_DN7483_c0_g2 *MYB* unigene showed that the R3 domain was incomplete; therefore, we searched the unfiltered original assembly sequence and found that this unigene had eight transcripts. Primers *FvPAP1*-F1 and *FvPAP1*-R1 (Table 1) were designed based on the common sequences at the 5′ and 3′ ends of these eight transcripts to amplify the longest complete CDS sequence of the gene. A total of 1% agarose gel electrophoresis of the amplified product showed a single band (Figure S4). After cloning to the T-vector, the sequencing showed that there were differences among the sequences of different clones. A total of 26 clones were sequenced. The sequencing results showed that the predicted protein sequences of fifteen clones had a complete R2R3 domain, two clones possessed an incomplete R2 domain, and the other nine clones had completely lost the R2R3 domain. A sequence alignment of the nucleotides showed that the deletion of the R2R3 domain in the protein sequence of the seven clones was owing to the insertion of a GCAA 4 base and a large number of deletions of 175 bp in the coding region, which resulted in coding shift changes and premature termination (Figure S5). In addition to these two obvious changes, there were also other indels and base substitutions at other positions of the gene. These results suggest that there may be selective splicing and/or sequence polymorphism at this locus. We registered the sequence T3—FvPAP1—44 (Figure S5), which encodes a gene with the complete R2R3 domain and is the closest to AtPAP2 in GenBank as *FvPAP1* (OR682444).

### 3.5. Analysis of the Expression of the Anthocyanin Biosynthetic and Regulatory Genes

**Figure 4:**
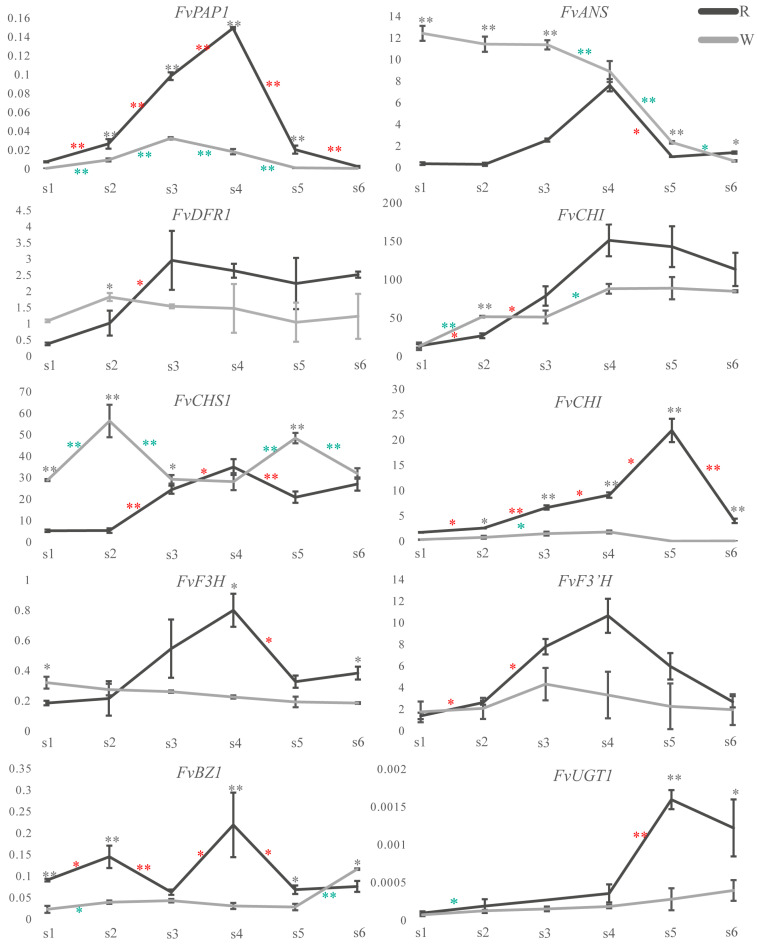
The levels of expression of the genes related to anthocyanin biosynthesis during the development of leaves in the W-type and R-type *Ficus virens* plants. A Student’s *t*-test was used to evaluate the significance of the difference between the adjacent stages. The annotation between periods indicates that there are differences between adjacent periods (W-Type * is red *, W-Type * is green *, * *p* < 0.05. ** *p* < 0.01), and the annotation directly above the period indicates the difference between materials (* is black *, * *p* < 0.05, ** *p* < 0.01).

To further verify the relationship between the changes in expression of the genes related to anthocyanin biosynthesis and content in the transcriptome sequencing results, a qRT-PCR analysis was performed on the six developmental stages of the W-type and R-type *F. virens* leaves. The results showed that the results of qRT-PCR were consistent with the trend of changes for each gene in the transcriptome data (Figure 4).

The expression of *FvPAP1* and the change in the content of anthocyanins in the leaves of W-type first increased and then decreased, but the anthocyanins reached their peak at s4, while the highest level of expression of *FvPAP1* was at s3. The levels of expression of *FvPAP1* were consistent with the trend in the changes of anthocyanin during the development of the R-type *F. virens* leaves, and both reached their highest values at the s4 stage (Figure 4).

The levels of expression of *FvPAP1*, *FvDFR1*, *FvCHS2*, and *FvF3*′*H* showed a trend of increasing first and then decreasing during the development of the W-type *F. virens* leaves. The change in the level of expression of *FvCHS2* was completely consistent with the trend in the change of anthocyanin content. Both reached their highest values at the s4 stage. The change in the level of expression of *FvF3*′*H* was consistent with the change in the level of expression of *FvPAP1*, which reached its highest value at the s3 stage. The levels of expression of *FvANS*, *FvDFR1*, *FvCHI*, *FvCHS1*, *FvF3H*, *FvBZ1*, and *FvUGT1* did not show the same tendency to vary in parallel with the changes in anthocyanin content or level of expression of *FvPAP1* (Figure 4).

During the development of the R-type *F. virens* leaves, the levels of expression of most genes showed a trend of increasing first and then decreasing. The trend of the change in *FvPAP1* expression was consistent with that in anthocyanin content, and the highest value appeared at the s4 stage. The trend of change in the levels of expression of the *FvCHI*, *FvCHS*, *FvANS*, *FvF3H*, and *FvF3*′*H* genes was consistent with that of the anthocyanin content and *FvPAP1* expression, and the highest value appeared at the s4 stage. The level of expression of *FvDFR1* reached its peak at the s3 stage, and the levels of expression of *FvCHS2* and *FvUGT1* reached their peak at the s5 stage. The levels of expression of these genes were consistent with the changes in anthocyanin content and *FvPAP1* on the whole. However, the levels of expression of *FvBZ1* formed two peaks at the s2 and s4 stages (Figure 4).

### 3.6. Functional Verification of FvPAP1

The level of expression of *FvPAP1* was basically consistent with the content of anthocyanin and the levels of expression of *FvF3*′*H*, *FvANS*, and *FvDFR1*. A phylogenetic tree analysis showed that *FvPAP1* clustered with the *MYB* protein (AtMYB75/PAP1, AtMYB90/PAP2, AtMYB113, AtMYB114, PhAN2, and PhAN4) that activates the biosynthesis of anthocyanin. These suggested that *FvPAP1* may be involved in the activation of anthocyanin biosynthesis in the leaves of *F. virens*. However, whether it can promote the biosynthesis of anthocyanin merits further experimental verification. Because the technology of analyzing gene function by transient expression and stable expression has not been established in *F. virens*, we chose to further verify the function of *FvPAP1* through heterologous expression.

**Figure 5:**
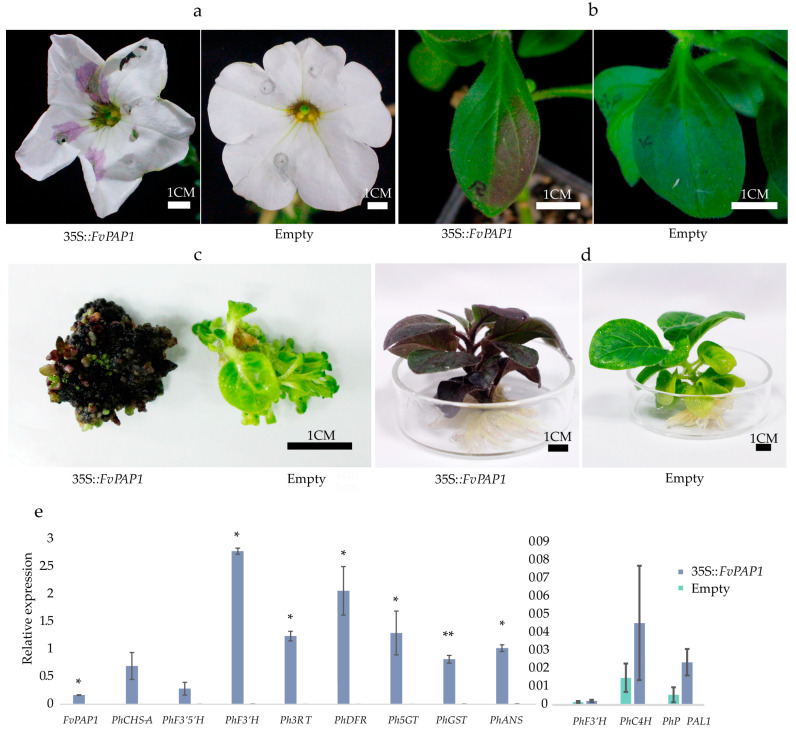
Functional analysis of *FvPAP1*: (**a**) Transient expression of *FvPAP1* in the *Petunia hybrida* flowers. (**b**) Transient expression of *FvPAP1* in the *P hybrida* leaves. (**c**) The purple phenomenon of *FvPAP1* in tobacco callus. (**d**) Purple phenomenon observed in tobacco seedlings after transformation with *FvPAP1*. (**e**) The levels of expression of the anthocyanin structural genes in the *P. hybrida* leaves that transiently expressed *FvPAP1*. The left side of each gene histogram is 35s::*FvPAP1* expression; the right side is empty expression, and both were calculated as the same internal gene reference. The significance was tested by a single-factor analysis of variance and t-test. * *p* < 0.05, ** *p* < 0.01.

First, we introduced the 35S:*FvPAP1* vector into the petals and leaves of petunia using the *Agrobacterium*-mediated method (injection) for transient expression. A total of 12 petunia petals were injected with 35S:*FvPAP1*, and seven of them began to show obvious purple spots at the injection site 3 days after injection (Figure 5a). Ten petals of *P. hybrida* were injected with the empty vector, and no purple spot appeared until the petals had wilted (approximately 7 days after injection). These results indicate that *FvPAP1* can activate the biosynthesis of anthocyanin in the floral organs.

A total of 27 *P. hybrida* leaves were injected with 35S:*FvPAP1*, and lavender spots appeared on the leaves (Figure 5b) on the fourth day after injection. Another 27 leaves of *P. hybrida* were injected with the empty vector, and no purple spot appeared within 14 days. On the ninth day after injection, the *P. hybrida* leaves were collected to extract the total RNA for qRT-PCR. The quantitative results showed that the levels of expression of the structural genes *PhCHSA*, *PhF3*′*5*′*H*, *PhF3H*, *PhDFR*, *PhANS*, *PhPAL1*, and *PhF3*′*H* in the anthocyanin biosynthetic pathway in the leaves injected with *FvPAP1* were significantly higher than those injected with the empty vector. These results indicate that the *FvPAP1* gene activates the biosynthesis of anthocyanin in the leaves (Figure 5e).

We subsequently used the leaf disc method to introduce *FvPAP1* into *Nicotiana tabacum*. A total of 97 resistant calli were obtained after transformation and screening with Basta, and 86 of them were dark purple-red, with obvious characteristics of anthocyanin accumulation. In addition, the adventitious buds that regenerated from the callus were purple-red. More than 100 resistant calli were obtained by transformation of the empty vector, and no purple calli were found (Figure 5c,d). These indicated that *FvPAP1* can stably activate the biosynthesis of anthocyanin in tobacco.

## 4. Discussion

With the increasing demand for landscape ecological construction, leaf color has attracted increasing amounts of attention because of its substantial ornamental value. The leaves of some woody plants appear red at the young leaf stage, and the red gradually fades with the development of the leaves. If the red phenomenon that occurs during the development of the leaves can be utilized, it will substantially enhance the landscape value. In *Ficus virens*, young buds germinate, and new leaves rapidly expand as soon at the old ones fall from the tree. The replacement of old leaves by new ones is completed in a very short time. The red color on the rapidly unfolding new leaves can provide a colorful scene at this stage and increase the visual effect to result in high ornamental values. We found that the R-type *F. virens* leaves maintained their red state for a longer time than the W-type in the new leaf developmental stage, and there was a corresponding improvement in the landscape effect.

The leaf color of plants is primarily determined by pigments. The detection of pigments in different developmental stages of the *F. virens* leaves showed that as the red color appeared and then disappeared with the development of leaves, the anthocyanin content increased first and then gradually decreased (Figure 1b). This result is consistent with research on the transiently flush young leaves that are red in *Castanopsis fiss* [37], *Syzygium luehmannii* [38], *Hevea brasiliensis* [39], *Juglans regia* [40], and other plants. The levels of expression of *FvCHI*, *FvANS*, *FvDFR1*, *FvF3*′*H*, and other structural genes related to anthocyanin biosynthesis were significantly higher in the red-leaf period than in the green-leaf period (s3–s5), which indicated that the red appearance of new leaves in *F. virens* was related to the change in the content of anthocyanin. The accumulation of anthocyanin during young leaf development is primarily considered as a defense measure against excess light in many plant species [41,42]. However, the specific role of anthocyanins in the development of young leaves remains to be elucidated.

The levels of expression of the structural genes during anthocyanin biosynthesis directly affects the anthocyanin content, and the MBW complex regulates the expression of the LBGs during the biosynthesis of anthocyanin. The change in the level of expression of *MYB* is key to the change in anthocyanin content. In tea (*Camellia sinensis*), the change in the level of expression of *MYB* is crucial for young red leaves [43]. The MBW complex of IbMYB1/IbMYB2/IbMYB3-IbbHLH2-IbWDR1 in *Ipomoea batatas* activated the expression of the structural genes *IbCHS-D* and *IbDFR-B* related to anthocyanin biosynthesis [44]. In this study, the level of expression of *FvPAP1* increased first and then decreased during the development of leaves in both W-type and R-type *F. virens*, which was consistent with the anthocyanin content. The level of expression of *FvPAP1* reached its highest level at the s3 stage in the WT, while it was highest at the s4 stage in the R-type. The level of expression in the trend of changes of *FvPAP1* was completely consistent with the trend in the change of anthocyanin content (Figure 4), which suggested an important role of *FvPAP1* in the formation of red leaves in *F. virens*.

To verify whether the level of expression of *FvPAP1* can promote anthocyanin biosynthesis, we constructed an overexpression vector, and investigated the function by transient expression in *P. hybrida* and stable expression in *N. tabacum*. The results showed that the transgenic tobacco callus was clearly purple, and the injection site in the petunia petals became purple. This phenomenon did not appear in the control material. These results verified that *FvPAP1* had the function of activating anthocyanin biosynthesis (Figure 5).

During the process of cloning *FvPAP1*, we found that the *FvPAP1* locus has sequence polymorphism. Only 57.6% of the cDNA sequence numbers had a complete R2R3 conserved domain, and the remaining cDNA sequences had some differences, which resulted in partial or complete deletion of the R2R3 domain. Because fragment deletion and indel did not occur in other genes cloned using the same experimental protocol and chemicals, it seems impossible that the sequence variation in the *FvPAP1* gene was caused by PCR amplification. These results suggest that there may be selective splicing and/or sequence polymorphism at this gene locus. The relationship between the duration of red-leaf period and *FvPAP1* polymorphism merits further study.

The prolonging of the red-leaf period is not only related to biosynthesis but also to the degradation of anthocyanin. There are various enzymatic and non-enzymatic factors that affect the stability and concentration of anthocyanin. Anthocyanin may become discolored as a result of active enzyme-driven breakdown processes. In addition to enzymatic factors, non-enzymatic factors (B-ring hydroxylation, glycosylation, metal ions, and pH, among others) and environmental factors (temperature, light, and irradiance among others) also affect the color and stability of anthocyanins and may enhance their vulnerability to the enzymes that degrade anthocyanins [21,45,46,47,48]. No significant differences in the levels of expression of the genes related to key genes of the light signaling pathway and anthocyanin degradation between the two varieties of *F. virens* were found during the process of transcriptome analysis in this study. The degradation of anthocyanin may not be the primary factor that causes the difference in the length of red-leaf period in *F. virens*.

Transcriptome sequencing technology has become an important tool to study the phenomena of plant life. However, owing to the lack of high-quality genome sequencing data in most plants, the de novo assembly of expressed sequences is still an important method for the analysis of DEGs and cloning of cDNA in plants. Owing to the complexity of many plant genomes, including a high degree of polyploidization and gene duplication, the de novo assembly of short NGS reads is very challenging, and errors easily occur in the assembled sequences. In this study, the *ANS* gene was not annotated in the results of Trinity assembly with default parameter. Thus, the putative expression boxes of *BZ1* (TRINITY_DN3563_c0_g1), *UGT* (TRINITY_DN13686_c0_g1), and *MYB* (TRINITY_DN7483_c0_g2) were not completely assembled. Primers were designed to amplify *CHS* (TRINITY_DN3608_c2_g1 and TRINITY_DN10900_c0_g1) and *F3H* (TRINITY_DN3755_c0_g1) based on the unigene sequences, but the assembled sequences were not obtained. This indicated that there were differences between the assembled and actual sequences. The *CHS* gene family may have highly expanded, which resulted in difficulty in sequence assembly because 21 *CHS* genes were annotated in the *F. microcarpa* genome, and only one complete CDS of *CHS* gene with a high level of expression was amplified after our substantial research in this study. Therefore, we believe that if a DEG analysis is conducted using NGS technology in a plant with a complex genome, it is necessary to clone the complete CDS sequence of the target gene to confirm whether the assembled sequence is correct before quantitative PCR amplification is performed.

## 5. Conclusions

The change in color in the red leaves of *Ficus virens* was primarily caused by the change in the content of anthocyanin. *FvPAP1* could stably activate the biosynthesis of anthocyanin. The difference in the level of expression of *FvPAP1* in time and intensity in the young leaves may be the reason for the difference in the duration of the red-leaf period in different lines of *Ficus virens*.

## Figures and Tables

**Table 1:** Primer sequence and application.

Primer	Primer Sequence	Application
*FvPAP1*-F	ATGGATGGCCGTTCCTCTG	Cloning
*FvPAP1*-R	TTAATTTCCTTGGTCGAGATCC	
*FvANS*-F	ATGGTGTTCTCAGAGGCTTC	
*FvANS*-R	TTATTTGGAAATTCCGTTGCTATG	
*FvCHI*-F	ATGTCTCCGATGACACTTACA	
*FvCHI*-R	TCAAACTGCCAACAGTTTCTT	
*FvCHS2*-F	ATGGCCTCTGTATACGAAATC	
*FvCHS2*-R	TTAATCAAGGGCAAGACTGTG	
*FvF3’H*-F	ATGCCTTCTGTCTCGATCATCCT	
*FvF3’H*-R	TTATGCTTGATATACGTGTTGGGC	
*FvUGT1*-F	ATGGCAAAATTTCACATCGCC	
*FvUGT1*-R	TTAACTATGATTTGCCAGCTCTTCC	
*FvF3H*-F	ATGTCTCCGCCTTCAACTCTC	
*FvF3H*-R	TTAAGCCAGAATCTGGTCGAGAG	
*FvDFR1*-F	ATGGTATCGGAGGGCGAGAT	
*FvDFR1*-R	CTAAGCCTCCACGCCATTG	
*FvBZ1*-F	ATGGCATCACCACCACCAGC	
*FvBZ1*-R	CTAAAACGTCGGACGATTCAACGG	
*FvCHS*-F	ATGGTGACTGTCGAGGAGGTC	
*FvCHS*-R	CTAGATTGCCACGCTATGGAGC	
*FvF3H*-QF	GGCAGTGGTGAACTCAAACTACAG	qRT-PCR
*FvF3H*-QR	CCTGGCAAGCTCAAGGTCCTT	
*FvANS*-QF	GCCCTCACCTTCATCCTACACAAC	
*FvANS*-QR	GGAGAATACTCTTGAACTTGCCATTGC	
*FvCHI*-QF	CACACAGTCACCATCCGGTTCCT	
*FvCHI*-QR	TTCCGCCAGAATTGAAGCCATGC	
*FvCHS1*-QF	TGGGAATCTCAGACTGGAACTCTCTT	
*FvCHS1*-QR	GGCACACTTCCTCCTCATCTCATC	
*FvCHS2*-QF	TGACGGGCATCTGAGGGAAGTAG	
*FvCHS2*-QR	TGGTCTCCACCTGATCCAGAATCG	
*FvDFR1*-QF	CCGTTACATCGCCAGTTCACAC	
*FvDFR1*-QR	CTGCTTCAACGAACATATCCTCCAAG	
*FvF3’H*-QF	GGAATGACTTTGAAGTGATACCGTTTGG	
*FvF3’H*-QR	GCCCGTTGTAGAGTGAGCCCAT	
*FvUGT1*-QF	GTGGATCTTTAATATCCTCCTCGGTTACC	
*FvUGT1*-QR	TTCCTCTTCGCCGCCAAACC	
*FvBZ1*-QF	GATCTCAAGTCCAAATTCAAGAAGTTCCTC	
*FvBZ1*-QR	ACCATGACCGAACCAAAGCTAATGT	
*FvPAP1*-QF	GCATGATTGGTACAAATACATCCGAGG	
*FvPAP1*-QR	CAGGTTGATCTTCAGCTAATTCTTGAGAC	
*FvACT*-QF	CCTCTACGGCAACATTGTCCTCAG	
*FvACT*-QR	CTCCGATCCAGACACTGTACTTCCT	
*FvUBi*-QF	CTCTCCACCTTGTCCTCCGTCTTC	
*FvUBi*-QR	CCCAAAGCAGCAACGACAACCAT	
*FvPAP1*-QF	GCATGATTGGTACAAATACATCCGAGG	
*FvPAP1*-QR	CAGGTTGATCTTCAGCTAATTCTTGAGAC	
*FvACT*-QF	CCTCTACGGCAACATTGTCCTCAG	
*FvACT*-QR	CTCCGATCCAGACACTGTACTTCCT	
*FvUBi*-QF	CTCTCCACCTTGTCCTCCGTCTTC	
*FvUBi*-QR	CCCAAAGCAGCAACGACAACCAT	

**Table 2:** Candidate genes for anthocyanin biosynthesis in *Ficus virens*.

Candidate Gene	Total No.	Unigene	Name	CDS Length	GenBank Accession Number
*ANS*	1	TRINITY_DN2320_c1_g1 (k-mer = 25)	*FvANS*	1077 bp	OR682438
*ANR*	1	TRINITY_DN11302_c0_g1	*-*	-	
*BZ1*	1	TRINITY_DN3563_c0_g1	*FvBZ1*	1104 bp	OR682439
*CHS*	5	TRINITY_DN7475_c0_g2	*-*	-	
		TRINITY_DN3608_c2_g1	*FvCHS1*	-	OR682437
		TRINITY_DN10900_c0_g1	*-*	-	
		TRINITY_DN7475_c0_g1	*-*	-	
		TRINITY_DN579_c1_g1_i1 (k-mer = 25)	*FvCHS2*	1173 bp	OR682441
*C4H*	2	TRINITY_DN4483_c0_g2	*-*	-	
		TRINITY_DN4483_c0_g1	*-*	-	
*F3*′*H*	1	TRINITY_DN13173_c0_g1	*FvF3H*	1530 bp	OR682443
*C3*′*H*	2	TRINITY_DN9414_c1_g1	*-*	-	
		TRINITY_DN8383_c0_g1	*-*	-	
*DFR*	2	TRINITY_DN16497_c0_g1	*FvDFR1*	1086 bp	OR682442
		TRINITY_DN2850_c0_g1	*-*	-	
*CAMT*	1	TRINITY_DN542_c1_g1	*-*	-	
*HCT*	4	TRINITY_DN2998_c0_g1	*-*	-	
		TRINITY_DN1799_c0_g1	*-*	-	
		TRINITY_DN755_c0_g2	*-*	-	
		TRINITY_DN29_c2_g1	*-*	-	
*CHI*	2	TRINITY_DN16071_c0_g1	*-*	-	
		TRINITY_DN998_c0_g1	*FvCHI*	702 bp	OR682440
*F3H*	1	TRINITY_DN3755_c0_g1	*FvF3H*	-	OR682435
*FLS*	1	TRINITY_DN3166_c1_g1	*-*	-	
*LAR*	2	TRINITY_DN10406_c0_g2	*-*	-	
		TRINITY_DN408_c0_g1	*-*	-	
*UGT*	1	TRINITY_DN13686_c0_g1	*FvUGT1*	1374 bp	OR682445
*MYB*	1	TRINITY_DN7483_c0_g2	*FvPAP1*	939 bp	OR682444

-: no gene cloning, the full length of the target sequence was not cloned.

## Data Availability

Data is contained within the article and Supplementary Material.

## Supplementary Materials

The following supporting information can be downloaded at: https://www.mdpi.com/article/10.3390/cimb46060343/s1, Figure S1: Red-type (R-type) leaves of *Ficus virens*; Figure S2: pGMF500 vector; Figure S3: Bud of *Ficus virens* is wrapped by a large stipule; Figure S4: Electrophoretogram of cloned gene. Marker is DL2000. Gene annotation * is expressed as the result of cloning using qRT-PCR primers. Electrophoretogram of qRT-PCR primers: The upper band was W-type *Ficus virens* as the template, and the lower band was R-type *Ficus virens* as the template. From left to right, the quantitative primers of *FvDFR*, *FvF3*′*H*, *FvCHS*, *FvPAP1*, *FvUGT1*, *FvANS*, *FvCHI*, *FvBZI*, and *FvF3H* were cloned, * indicates that the gene is a product of qRT-PCR primer cloning; Figure S5: *FvPAP1* polymorphism: (a) Amino acid sequence of R2R3 domain of *FvPAP1*. (b) Partial sequence of *FvPAP1.* Red arrow represents the insertion of a GCAA 4 base.
