# Supplementary figures and images for: Transcriptome Analysis Reveals That FvPAP1 Genes Are Related to the Prolongation of Red-Leaf Period in Ficus virens
Source: Curr Issues Mol Biol. 2024 Jun 8;46(6):5724–43. doi: 10.3390/cimb46060343 (PMC11202158; doi:10.3390/cimb46060343)

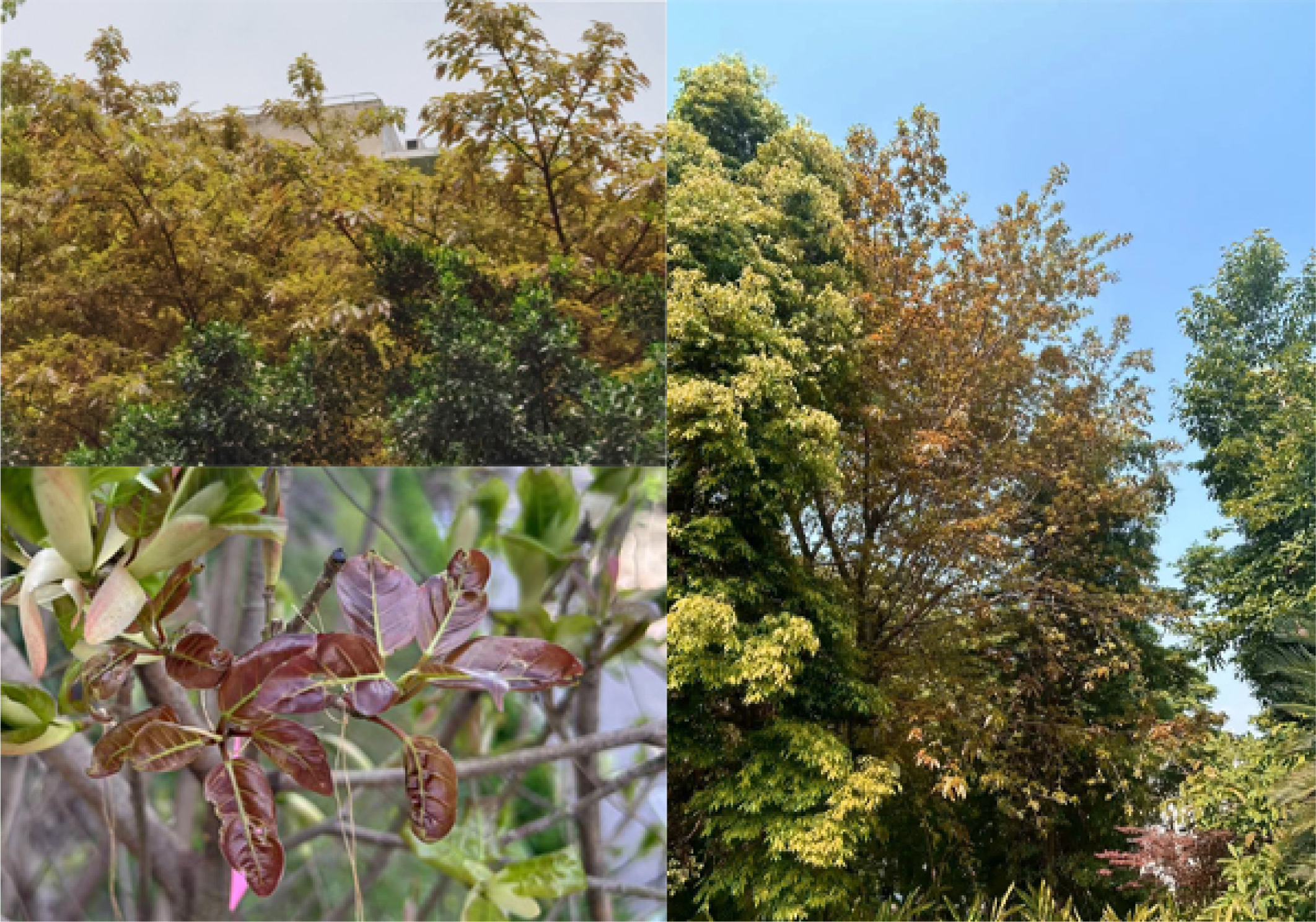

Supplement: Supplementary file 1 [file cimb-46-00343-s001.zip › Figure S1. Red-type (R-type) leaves of Ficus virens.tif]

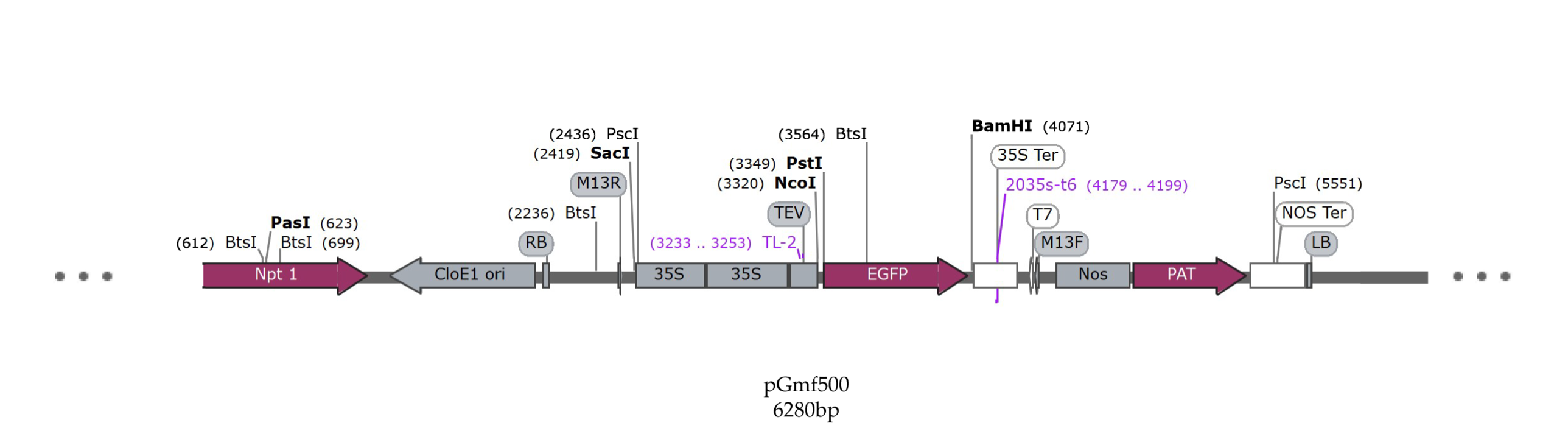

Supplement: Supplementary file 1 [file cimb-46-00343-s001.zip › Figure S2. pGMF500 vector.tif]

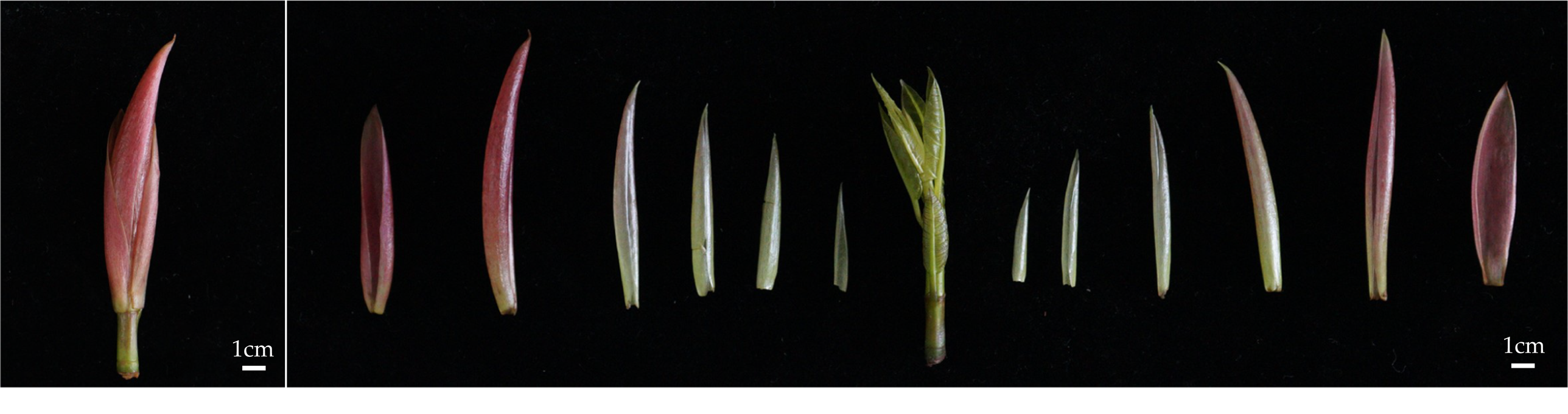

Supplement: Supplementary file 1 [file cimb-46-00343-s001.zip › Figure S3. Bud of Ficus virens is wrapped by a large stipule.tif]

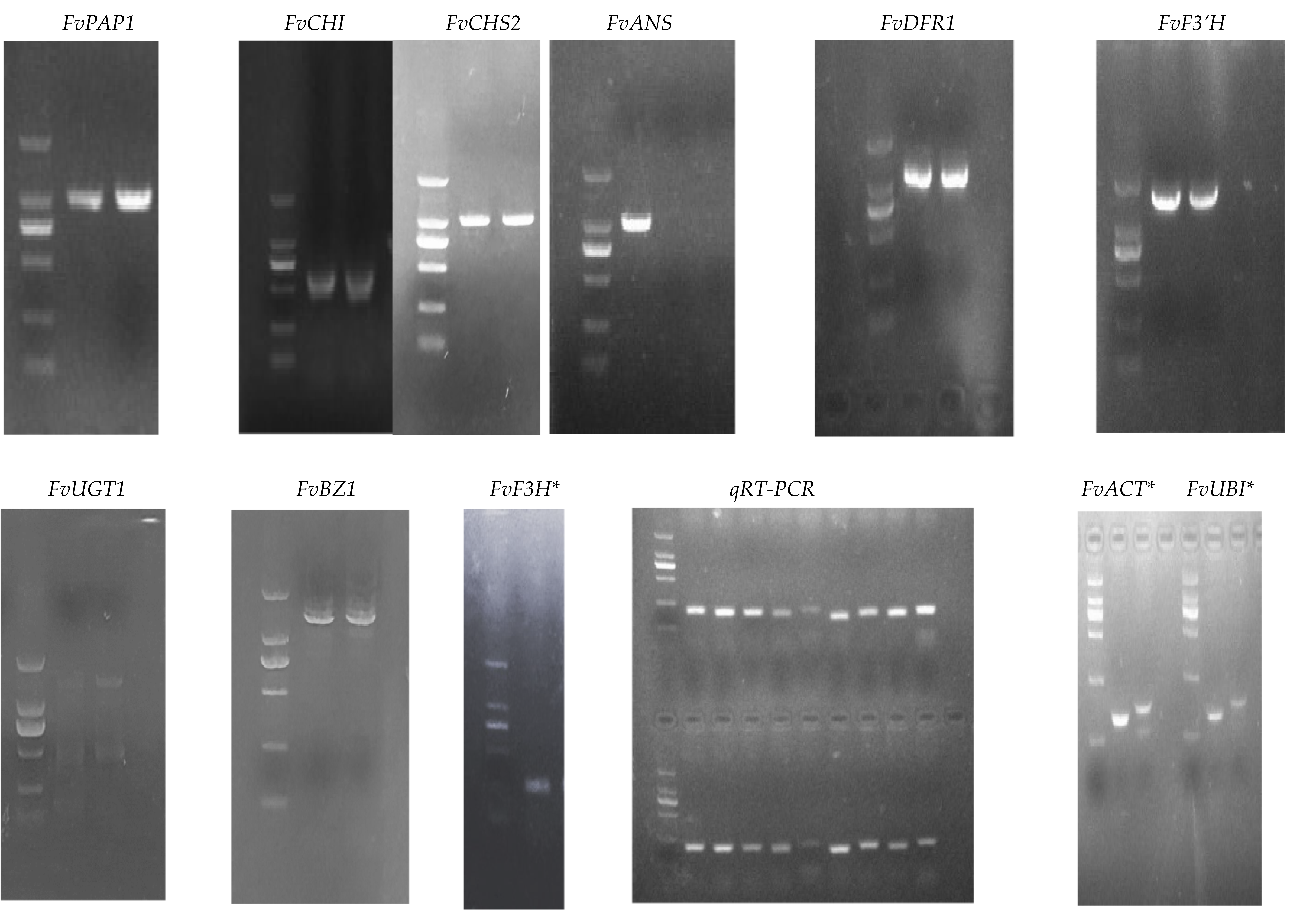

Supplement: Supplementary file 1 [file cimb-46-00343-s001.zip › Figure S4. Electrophoretogram of cloned gene.tif]
